# Management of shoulder instability in hypermobility-type Ehlers-Danlos syndrome

**DOI:** 10.1016/j.xrrt.2021.03.002

**Published:** 2021-03-24

**Authors:** Samuel E. Broida, Aidan P. Sweeney, Michael B. Gottschalk, Eric R. Wagner

**Affiliations:** Department of Orthopaedic Surgery, Emory University, Atlanta, GA, USA

**Keywords:** Shoulder instability, hypermobility, multidirectional instability, Ehlers-Danlos syndrome, shoulder arthroscopy, recurrent instability

## Abstract

Shoulder instability in hypermobile Ehlers-Danlos syndrome can result in lifelong pain and functional disability. Treatment in this population is complicated by the severe degree of instability as well as the underlying abnormalities of the joint connective tissue. Appropriate care for these patients requires a thorough understanding of the natural history of their disease, knowledge of the available treatment options, and certain special considerations. This article reviews the pathoanatomy, recognition, and management of shoulder instability in the patient with hypermobile Ehlers-Danlos syndrome.

Ehlers-Danlos syndrome (EDS) refers to a group of heritable connective tissue disorders that result from underlying defects in collagen formation. While the true prevalence of EDS is unclear, estimates vary from a rate of 1 in 500 to 1 in 20,000 people.^14^^,^^19^^,^^73^ Clinical features of EDS vary broadly across the 13 recognized subtypes, making timely diagnosis difficult for nonspecialists (Table I).^12^^,^^52^ Common phenotypic hallmarks include hypermobile joints, hyperextensible skin, and fragile connective tissue.^12^^,^^52^ These features often manifest as musculoskeletal complaints such as pain and joint instability. Joint instability is commonly seen in the classical and hypermobile EDS types, which account for 90% of total cases.^81^ A multidisciplinary approach to the patient with EDS is critical, including physiatrists, physiotherapists, occupational therapists, and orthopedic surgeons.^23^ For these specialists and patients, recurrent joint instability is perhaps the most challenging musculoskeletal feature of EDS, given that the resultant pain and loss of function can be both debilitating and refractory to most standard therapies.

**Table I:** Clinical manifestations of the thirteen Ehlers-Danlos syndrome clinical subtypes.

EDS subtype	Abbreviation	Major and minor clinical features
Classical (AD)	cEDS	Major: Hyperextensile skin, atrophic scarring, joint hypermobility
		Minor: Easy bruising, molluscoid pseudotumors, recurrent hernias
Classical-like (AR)	clEDS	Major: Hyperextensile skin, easy bruising, joint hypermobility
		Minor: Foot deformities, hand/finger deformities, axonal polyneuropathy
Hypermobile (AD)	hEDS	Major: Generalized joint hypermobility
		Minor: Mild skin hyperextensibility, soft/velvety skin, mild atrophic scarring
Vascular (AD)	vEDS	Major: Excessive bruising, arterial fragilitiy/rupture, characteristic facies
		Minor: Arteriovenus fistula formation, spontaneous pneumothorax, acrogeria
Cardiac-valvular (AR)	cvEDS	Major: Severe valvular disease, hyperextensile skin, joint hypermobility
		Minor: Foot deformities, pectus deformity, hernia
Arthrochalasis (AD)	aEDS	Major: Hyperextensile skin, atrophic scarring, joint hypermobility
		Minor: Easy bruising, muscular hypotonia, kyphoscoliosis
Dermatosparaxis (AR)	dEDS	Major: Severe skin fragility, redundant skin, characteristic facies
		Minor: Growth deficiency, delayed gross motor development, palmar wrinkling
Kyphoscoliotic (AR)	kEDS	Major: Congenital hypotonia, early onset kyphoscholiosis, hypermobility
		Minor: Hyperextensile skin, easy bruising, marfanoid habitus, blue sclerae
Brittle cornea syndrome (AR)	BCS	Major: Thin cornea, early onset keratoconus & keratoglobus, blue sclerae
		Minor: Retinal detachment, deafness, scoliosis, DDH, arachnodactyly
Spondylodysplastic (AR)	spEDS	Major: Short stature, muscle hypotonia, limb bowing
		Minor: Hyperextensile skin, delayed psychomotor development, osteopenia
Musculocontractural (AR)	mcEDS	Major: Congenital contractures, characteristic facies, hyperextensile skin
		Minor: Axial and limb deformities, GI and GU abnormalities, ocular disease
Myopathic (AD or AR)	mEDS	Major: Congenital hypotonia/atrophy, proximal joint contractures, hypermobility
		Minor: Atrophic scarring, delayed gross motor development, myopathy
Periodontal (AD)	pEDS	Major: Severe periodontitis, absent gingiva, pretibial plaques
		Minor: Easy bruising, hypermobility, hyperextensile skin, marfanoid facies

*AD*, autosomal dominant; *AR*, autosomal recessive.

Joint dislocations occur in around 75% of all patients with EDS.^80^ Instability may be seen in most clinical EDS subtypes (classic, classic-like, hypermobile, cardiac valvular, EDS/osteogenesis imperfecta overlap syndrome, arthrochalasia), though patients with hypermobile-type EDS (hEDS) are particularly prone. More than 95% of people with hEDS report joint dislocations, and the majority opt for surgical treatment at some point in their lifetime.^12^^,^^83^ While instability of almost every joint has been described, the shoulder appears to be among the most commonly affected joints.^12^^,^^70^ This is owing to the lack of bony stability within the glenohumeral joint and instead a reliance on dynamic and static soft tissue stabilizers. In EDS, the static soft tissue stabilizers are deficient, and the dynamic stabilizers and intrinsic bony stability are insufficient to prevent subluxations and dislocations. In this article, we will review the recognition and management of shoulder instability in the patient with hEDS.

## Diagnosis

The diagnosis of hEDS must be made clinically as there are currently no conclusive genetic markers for this subtype.^26^ As per the 2017 International Classification of Ehlers-Danlos Syndromes,^52^ three clinical criteria must be fulfilled to establish the diagnosis of hEDS (Table II). The first criterion is generalized joint hypermobility based on the Beighton scoring system with appropriate age- and sex-adjusted cutoffs (Table III).^6^ The second criterion is fulfillment of at least two of the following: positive family history for hEDS, pain and secondary musculoskeletal complications of joint laxity, and systemic manifestations associated with heritable connective tissue disease.^52^ The final criterion is an exclusion of other possible spontaneous and genetic causes of joint hypermobility.

**Table II:** Requisite criteria for diagnosis of hEDS.

1. Generalized joint hypermobility a. Prepubertal and adolescent: Beighton ≥ 6 b. Pubertal men and women of age 50 yr and younger: Beighton ≥ 5 c. Men and women older than 50 yr: Beighton ≥ 4
2. At least two of the following features: a. Systemic manifestations of a generalized connective tissue disease b. Positive family history with 1+ first-degree relatives meeting criteria for hEDS c. One or more of the following musculoskeletal complications: i. Daily musculoskeletal pain in ≥2 limbs for at least 3 mo ii. Chronic, widespread pain for at least 3 mo iii. Recurrent atraumatic joint dislocations or frank joint instability, defined by 3+ atraumatic dislocations in a single joint or 2+ atraumatic dislocations in different joints at different times
3. All of the following features: a. Absence of unusual skin fragility b. Exclusion of other hertiable and acquired connective tissue disorders c. Exclusion of alternative diagnoses that may also include hypermobile joints

*hEDS*, hypermobile-type Ehlers-Danlos syndrome.

**Table III:** Beighton laxity score.

Feature	Points
1. Passive dorsiflexion of 5th finger beyond 90º	0: unable, 1: unilateral, 2: bilateral
2. Passive apposition of thumb to flexor aspects of the forearm	0: unable, 1: unilateral, 2: bilateral
3. Hyperextension of the elbows beyond 10º	0: unable, 1: unilateral, 2: bilateral
4. Hyperextension of the knees beyond 10º	0: unable, 1: unilateral, 2: bilateral
5. Ability to easily rest palms on the floor with forward flexion of the trunk, knees straight, and feet together	0: unable, 1: able

## Pathoanatomy

**Figure 1:**
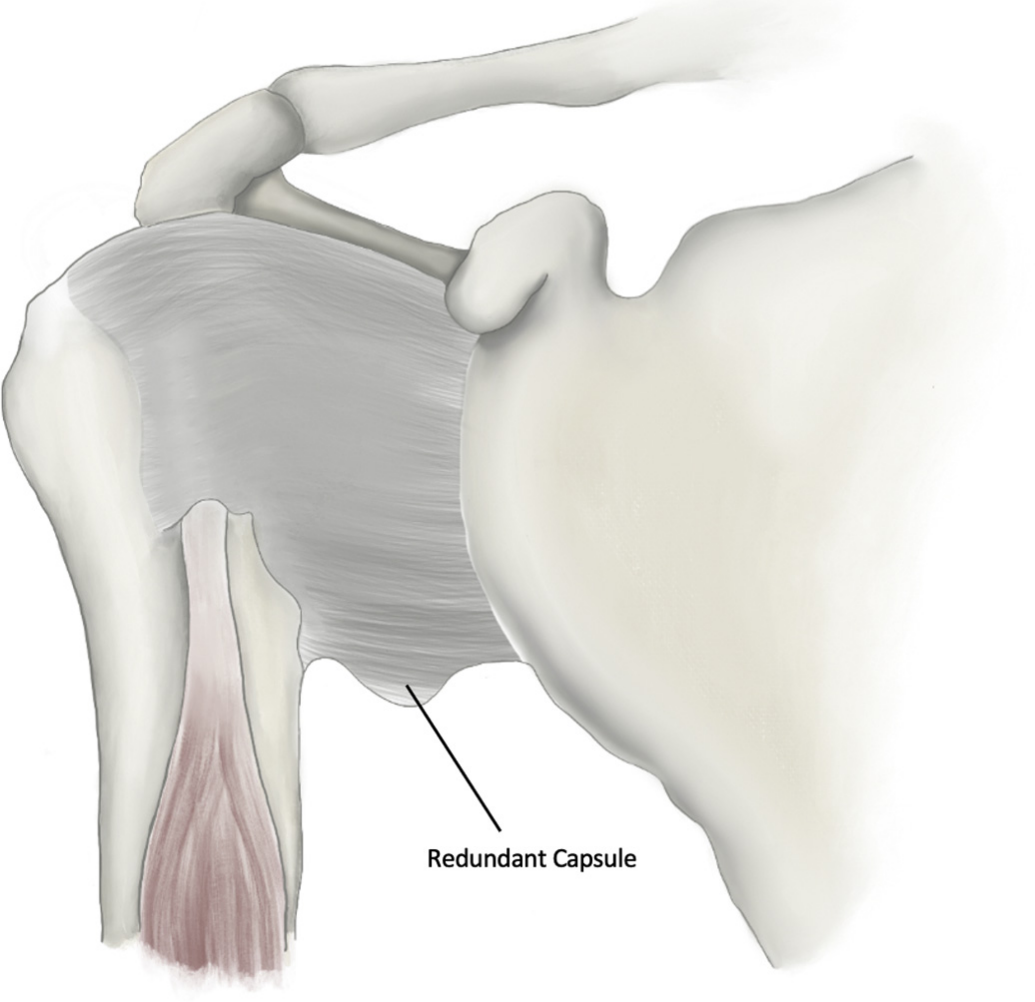
Illustration of capsular redundancy of the glenohumeral joint as seen commonly in hEDS. *hEDS*, hypermobile-type Ehlers-Danlos syndrome.

To understand pathophysiologic shoulder instability in hEDS, it is important to first appreciate the stabilization mechanisms of the healthy shoulder. In the normal shoulder, glenohumeral joint stability is dependent on adequate glenoid bone stock as well as static and dynamic stabilizers. The static stabilizers include the superior glenohumeral ligament; middle glenohumeral ligament; the anterior, posterior, and superior bands of the inferior glenohumeral ligament; the joint capsule; and the rotator interval, which contains the coracohumeral ligaments. The dynamic stabilizers of the shoulder include the four rotator cuff muscles and long head of the biceps, as well as the deltoid and periscapular muscles, to a lesser extent. The joint capsule is normally relatively loose and redundant to permit a wide range of multidirectional (>270°) motion. However, the patient with hEDS will have an especially capacious and expanded capsule with very little static stability (Fig. 1).^60^^,^^74^ This capsular and ligamentous laxity can often lead to low-energy subluxations or dislocations. Thus, a patient presenting with low-energy dislocation events—or even the ability to self-subluxate—should raise suspicion for hEDS, especially if accompanied by complaints of other unstable extremity or axial joints, skin hyperextensibility, atrophic scarring, or chronic joint pain.^12^

Chronic and recurrent subluxation can result in asymmetric loading of the joint. The hypermobile joint in EDS becomes painful often well before any visible changes can be detected on plain radiographs. Patients may also present with distal upper extremity neuropathic pain owing to traction or compression of the peripheral nerves.^23^ Poor muscle tone and impaired proprioception throughout development can contribute to immature postural reactions, and thus, people with hEDS may demonstrate signs of postural instability.^16^^,^^22^^,^^30^^,^^69^

Many patients with hEDS will develop scapular dyskinesia, either as a compensatory mechanism for the recurrent subluxations/dislocations or contributing to them. Furthermore, patients occasionally have voluntary or involuntary muscle contractions affecting their recurrent instability sensations. For example, latissimus dorsi contractions are associated with recurrent posterior and inferior humeral head subluxation. Years of pain avoidance is often the driving force underlying many of these altered mechanics.

## Natural history

Castori et al^13^ described three distinct phases in the natural history of hEDS.

### Phase 1: hypermobility

The first phase, labeled the “hypermobility” phase, is characterized by marked ligamentous laxity and may begin shortly after birth. If present, clinical signs of hEDS in the newborn are typically limited to the hip joint as unilateral or bilateral congenital dislocations. Shoulder hypermobility may present as early as childhood with reports in children as young as 5 years of age.^58^ This therefore poses a diagnostic challenge at young ages, as children are inherently more flexible than adults.^12^^,^^83^ Although distinction between a normal flexible child and one with mild hEDS can be difficult to discern, those with more severe disease can contort and bend into unusual positions through voluntary joint subluxations. Subsequently, children with hEDS have a predilection for sports requiring flexibility, such as ballet and gymnastics, and are able to participate without much functional disability. The most common clinical complaint during this first phase is joint instability and recurrent dislocations, especially of the patella.

### Phase 2: pain

The second phase is the “pain” phase. This phase typically starts during the second to fourth decades of life. Progressive and generalized musculoskeletal pain begins to hinder the patient with hEDS. Typically, there is a slight decrease in joint hypermobility compared with the first phase, with Beighton scores^6^ still remaining >4. Muscle, joint, and back pain are accompanied by worsening fatigue and may be mislabeled as fibromyalgia or psychosomatic symptoms. Combined with joint instability, these complaints cause progressive limitation in physical activity and activities of daily living. Compensation for unstable and painful joints may contribute to muscular atrophy, deconditioning, and alterations in joint mechanics which further exacerbate chronic pain. For example, in the shoulder, patients will often develop either scapular winging or dyskinesia as a compensatory mechanism for glenohumeral instability. Furthermore, they might develop voluntary or involuntary muscle contractions that contribute to the instability episodes, such as latissimus dorsi contractions associated with recurrent posterior and inferior instability.

### Phase 3: stiffness

Transition to the third and final phase, the “stiffness” phase, may occur in late adulthood. Considerable generalized joint hypermobility yields to joints that are slightly stiffer yet still prone to dislocation, with a gradual lowering of the Beighton score. This is likely due to decades of overuse and hypertonia of the dynamic joint stabilization muscles as they compensate for soft-tissue laxity. As these patients age and the shoulders stiffen, they are still prone to dislocation. However, their dislocations become more worrisome owing to associated risk of rotator cuff injuries and fractures secondary to stiffness. These patients continue to be plagued by pain as scapulothoracic dyskinesia worsens and becomes more symptomatic. Physical deformities such as kyphoscoliosis may also worsen. The patient with hEDS in this phase of illness is often significantly disabled secondary to pain, fatigue, limited range of motion (ROM), a lifelong history of injury, and reduced muscle mass.^13^^,^^83^

## Physical examination

### Inspection

Inspection of the shoulder at rest and with active range of motion is key in the evaluation of hEDS. Signs of compensation for chronic instability include scapular dyskinesia associated with a protracted scapular resting state and voluntary or involuntary muscle contractions, as well as glenohumeral subluxations with any attempted active motion. Particular attention should be paid to the scapulothoracic kinetics, as patients may have dyskinesia from poor mechanics or scapular winging from hyperlaxity or nerve injuries.^78^ Years of pain avoidance and altered mechanics can cause a significant reduction of muscle mass, including visible atrophy of various muscles around the scapulohumeral shoulder girdle.

### Palpation

**Figure 2:**
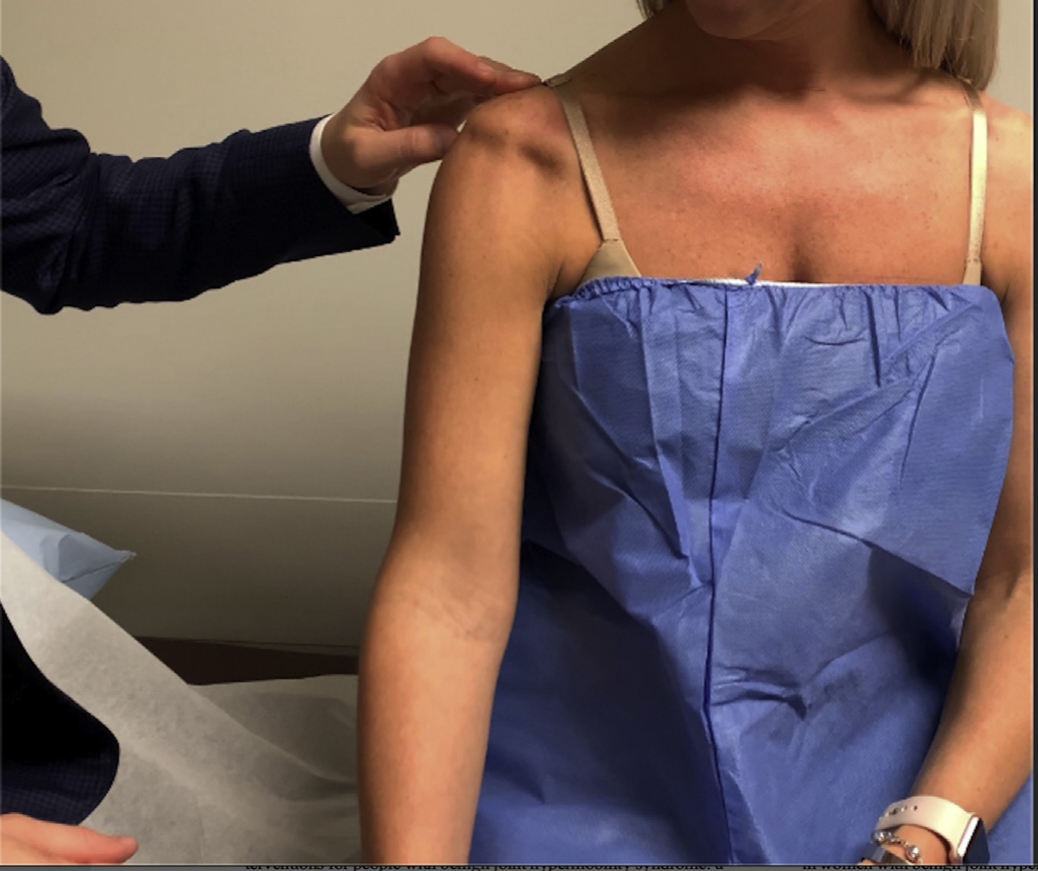
Demonstration of positive sulcus sign in a patient with hEDS. *hEDS*, hypermobile-type Ehlers-Danlos syndrome.

Instability of the shoulder in hEDS is usually multidirectional. On physical examination, there is increased translation of the humeral head beyond the glenoid rim in ≥2 directions with the arm resting in adduction at the side, termed multidirectional instability (MDI). The sulcus sign is also important to recognize, with inferior translation of the humeral head associated with a visible “sulcus” under the acromion (Fig. 2).

### Range of motion

Passive shoulder range of motion is often difficult to assess owing to apprehension and ease of dislocation. Active ROM of the shoulder may be above average or restricted by pain.^3^^,^^45^^,^^78^ Patients also may demonstrate signs of joint subluxation during active elevation or abduction. Many times, altered scapulohumeral rhythm can manifest with scapular dyskinesia during shoulder elevation movements. Although nearly all patients with hEDS endorse a history of joint laxity, only two-thirds of patients may demonstrate shoulder hypermobility at the time of physical examination.^13^ This is likely owing to delays in diagnosis and progressive articular stiffness that can develop from recurrent shoulder dislocations.

If hEDS is suspected, it is important to evaluate for hypermobility in other joints in addition the shoulder. Mean Beighton score for hEDS has been reported to be around 5.9 (compared with <2 in healthy populations) but is often greater than 7, 8, or 9 in adolescence and early adulthood.^6^^,^^79^

### Strength testing

Similar to ROM, strength testing is frequently limited by apprehension and significant instability. However, these patients often have poor rotator cuff tone and generalized shoulder weakness. This weakness is often most pronounced with shoulder abduction owing to deltoid atrophy. Certain scapular dyskinetic patterns will also alter the patients overall strength.

### Special testing

Anterior provocative maneuvers are often positive, including the load-and-shift test, apprehension test (abduction and external rotation), and relocation test.^84^ The posterior provocative maneuvers are also positive, including the posterior load-and-shift test and the Kims test.^24^^,^^44^ Although many of these maneuvers assess laxity of the glenohumeral joint, true instability may only be diagnosed if apprehension and discomfort are also present with testing.^35^ By the time of presentation, biplanar or triplanar instability in the patient with hEDS is often present in the midranges of shoulder motion rather than solely at the extremes.^3^

## Diagnostic imaging

**Figure 3:**
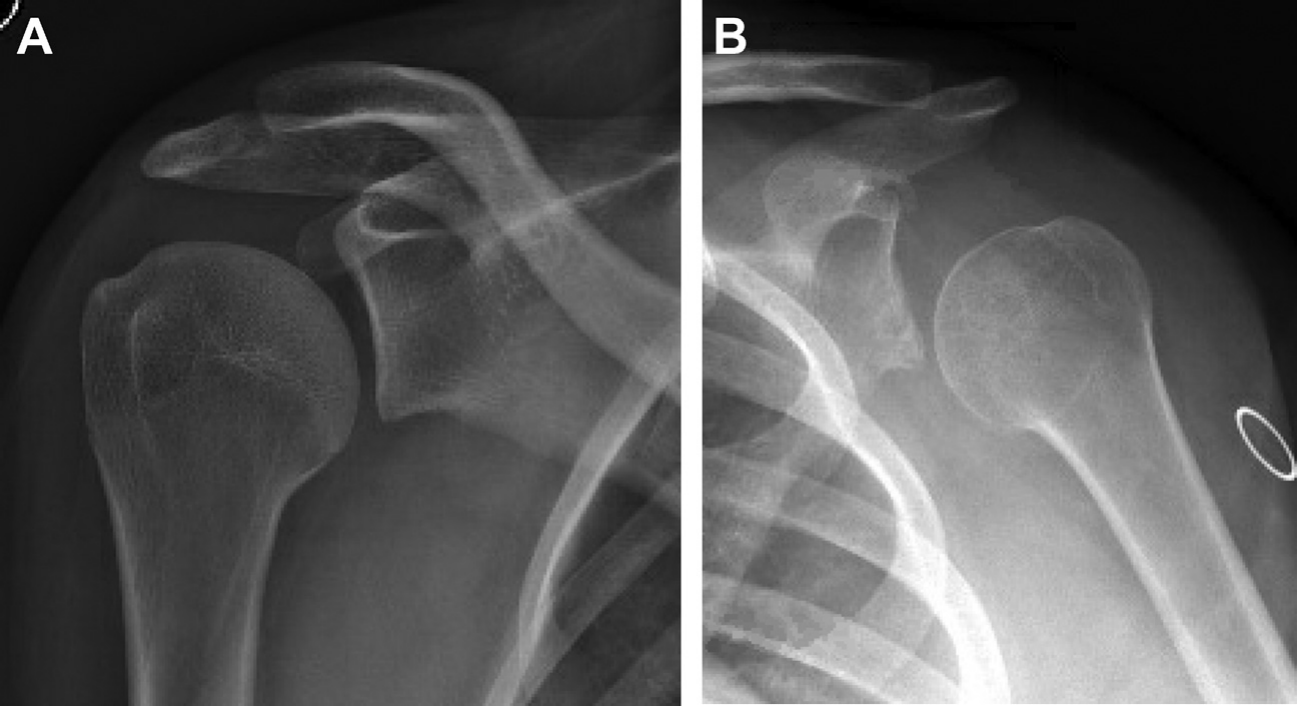
Radiographic demonstration of inferior subluxation of the humeral head in hEDS on Grashey views in patients with mild (**A**) and severe capsule laxity (**B**), indicating poor capsular integrity.

Imaging findings are variable in the patient with hEDS and depend on age at presentation and progression of symptoms. Static radiographs may show varying degrees of inferior humeral head subluxation relative to the severity of capsular laxity (Fig. 3). Given that instability in this population is owing to insufficiency of static stabilizers (eg, capsular insufficiency) rather than bony anatomy, static imaging with radiographs and computed tomography often do not demonstrate the underlying etiology for the instability. However, some patients with hEDS have glenoid dysplasia, best appreciated on Grashey views as a hypoplastic glenoid neck and on axillary views with glenoid retroversion. In these cases, a computed tomography scan of the shoulder can be useful for measuring glenoid version, medialization of the joint line, or recognizing other bony deficiencies. A longstanding history of subluxation and dislocation may eventually give way to glenohumeral erosion later in adulthood, but younger patients have loose, painful shoulder joints without changes in their labrum or bony anatomy.^23^^,^^35^

**Figure 4:**
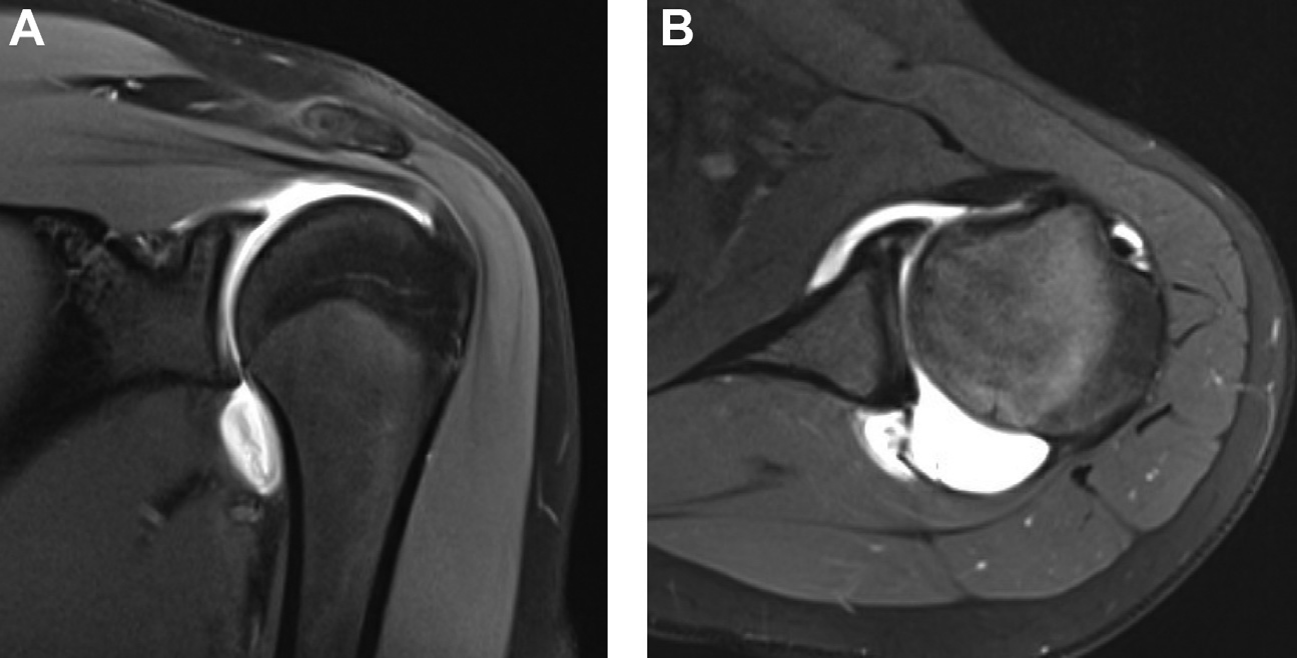
Magnetic resonance arthrography demonstrating capsular redundancy in a patient with hypermobile Ehlers-Danlos syndrome on coronal (**A**) and axillary (**B**) views.

Magnetic resonance imaging, both with and without intra-articular contrast, does provide some utility in patients with EDS with hypermobile shoulders. Standard magnetic resonance imaging of the shoulder may demonstrate soft-tissue findings consistent with connective tissue disease including a redundant capsule.^35^ Magnetic resonance arthrography either with saline or with contrast^77^ has an advantage in this regard, as it is a more sensitive modality in detection of capsular laxity and redundancy (Fig. 4). The particular finding on magnetic resonance arthrography for this pathology is an increase in the glenocapsular ratio, which is supportive of a diagnosis of hEDS.^61^ In contrast to healthy patients with traumatic dislocations, the absence of a labral tear on cross-sectional imaging of an unstable shoulder is often more suggestive of a diagnosis of underlying hEDS.

## Conservative management

As with MDI in patients without collagen defects,^53^ the first line and mainstay of treatment for shoulder instability in hEDS is rehabilitation and activity modification.^39^ Most patients who receive physiotherapy report an overall positive effect.^70^ The goal of rehabilitation in EDS is to achieve dynamic stability during activities of daily living and physical activity, focusing on increasing active rather than passive ROM.^79^ It is also critical to improve scapulohumeral coordination and correct any underlying scapular dyskinesia. Strengthening the resting tone of the rotator cuff, periscapular stabilizers, and deltoid is a priority in patients with hEDS. An imbalanced rotator cuff and deltoid force couple system can result in subacromial compression and impingement.^23^^,^^59^ Furthermore, a focus solely on the deltoid and rotator cuff without attention to the scapular stabilizers will often increase the scapular dyskinesia that many of these patients experience.

An emphasis on dynamic kinetics and controlled strengthening is essential for achieving functional stability of the glenohumeral and scapulothoracic articulations in patients with hEDS (Table IV). A recent observational study investigated humeral head translation with different isometric exercises in female patients with hypermobility spectrum disorder (including 13 women with hEDS) compared with healthy controls.^79^ They demonstrated an increase in acromiohumeral distance with weighted (2 kg) isometric external rotation and a decrease in acromiohumeral distance with weighted isometric shoulder flexion and shoulder/elbow extension. These results imply that physical therapy protocols in the patient with hEDS should focus on isometric external rotation for improvement in impingement symptoms and isometric forward flexion and extension for posterior-inferior instability. Similarly, Kitagawa et al described the case of an adolescent with hEDS with severe bilateral shoulder instability who experienced improvement in her symptoms through improvement in active ROM.

**Table IV:** Critical therapy components for multidirectional instability of the shoulder.

Exercise	Issue addressed
Isometric shoulder flexion	Posterior-inferior instability
Isometric shoulder and elbow extension	Inferior instability
Eccentric deltoid strengthening	Anterior instability
Eccentric rotator cuff strengthening, scapulothoracic and scapulohumeral retraining	Scapulothoracic dyskinesia
Postural and proprioceptive training	Postural instability
Isometric external rotation	Subacromial impingement

A large barrier to successful conservative treatment in EDS is recognition and treatment of patient discomfort. A survey of 78 patients with hEDS found that 90% of participants used analgesic medications, most commonly paracetamol-based drugs (65.4%) and non-steroidal anti-inflammatory drugs (42.3%).^18^ Poor localization, chronicity of pain, and delays in diagnosis may result in years of underprescription of analgesics and relaxants for patients with hEDS. However, there is a delicate balance between providing sufficient pain relief and limiting long-term dependence on these medications. Appropriate pain control through NSAIDs, muscle relaxants, neuropathic pain medications, and cannabinoids should allow the patient to complete activities of daily living and serve as a bridge for participation in physical therapy. Pain relief in patients with hEDS is often multimodal and therefore can be best approached by recruitment of a pain management specialist, especially if considering treatment with opioids or benzodiazepines. Additionally, much of the myofascial pain in hEDS may be secondary to muscle spasm as a result of chronic joint instability. Modalities commonly used to target muscle spasm include myofascial release, massage, electrotherapy, and heat therapy. The goals of these treatments are to afford temporary relief and allow for participation in strengthening and toning exercises for shoulder joint stability.^49^^,^^71^

There may also be some benefit to psychological evaluation and psychotherapy in some patients with hEDS, especially those who voluntarily self-dislocate for secondary gain.^38^ Prevalence of anxiety-related disorders among cohorts of patients with hEDS is high, ranging between 60 and 70%.^11^^,^^31^^,^^54^ García Campayo et al^31^ also noted that there was a significant correlation between the degree of joint hypermobility and severity of panic disorder. For this reason, it is important for providers to understand psychological barriers to treatment of patients with severe hEDS and arrange for appropriate care.

## Surgical treatment

Surgical management in this population is challenging owing to abnormalities of the connective tissue. The underlying abnormal collagen in hEDS results in structural insufficiency of the skin and other soft tissues. Fragile blood vessels and mild platelet deficiencies lead to more difficult hemostasis, increasing risk of intraoperative bleeding and postoperative hematoma.^5^ Furthermore, fibroblast dysfunction causes delayed healing for surgical incisions which may lead to wound complications.^56^^,^^71^^,^^89^ Repairs used in management of the otherwise healthy patient with shoulder instability may require additional augmentation in the EDS patient. Patients with unrecognized or improperly managed EDS will often present after multiple unsuccessful surgeries, including prior attempted Bankart or capsulolabral repairs, capsular reconstructions, capsulorrhaphies, and débridements.^75^ Soft tissue–only reconstructions using the patient’s native tissues have a high risk of failure in these patients owing to the inherent redundancy and insufficiency of these structures.^8^^,^^72^^,^^82^^,^^86^

As a result of these challenges, multiple surgical techniques have been proposed to treat shoulder instability in hEDS. These approaches are focused on the correction of capsular laxity with augmentation of the ligamentous or bony structures to compensate for native tissue insufficiency. In this section, we will review the various open and arthroscopic treatments for shoulder instability in hEDS, as well as the considerations for each.

### Arthroscopic capsulorrhaphy and Bankart procedure

Treatment of MDI with arthroscopic capsulorrhaphy has produced promising results in multiple cohorts of patients without known connective tissue disease.^32^^,^^43^^,^^67^ Application of this technique to hEDS is largely unreported in the literature, though Galano et al^29^ presented the case of a 16-year-old girl with hEDS who had resolution of shoulder instability at 21 months after arthroscopic capsular plication.

Open and arthroscopic Bankart repair have been widely studied and both are considered to be effective procedures for the treatment of recurrent anterior shoulder instability, particularly in those with glenoid labral tears.^9^^,^^25^^,^^66^ While the superiority of open vs. arthroscopic Bankart procedure is still a matter of debate,^28^^,^^33^^,^^51^ both approaches carry an increased risk of failure in hypermobile patients owing to their compromised soft-tissue integrity.^8^^,^^82^^,^^86^ Although the minimally invasive nature does not limit options for potential revision procedures, inadequate reinforcement of the weak underlying soft tissue in hEDS is a notable concern for these treatments.

### Thermal capsulorrhaphy

Thermal-assisted capsulorrhaphy has been presented as a less-invasive alternative to the Neer inferior capsular shift in treatment of shoulder instability. Radiofrequency energy is delivered arthroscopically through a monopolar thermal probe to the synovial surface of the redundant tissue, which denatures the collagen within the tissue and causes a lasting contraction of the joint capsule.^36^ In human cadaver and animal models, thermal capsulorrhaphy results in an initial impairment of tissue biomechanical properties and may further weaken the joint temporarily. However, the mechanical strength and stiffness of the tissue normalizes months after the procedure.^36^^,^^40^^,^^55^

A systematic review comparing arthroscopic capsular plication to thermal capsulorrhaphy included four studies involving 112 shoulders with MDI treated via thermal capsular shrinkage.^68^ Reported success rates ranged from 53% to 93% with a cumulative successful return to activity in 81% of patients, though postoperative protocols varied markedly with respect to duration of immobilization and initiation of active ROM. Despite these initially promising results, thermal capsulorrhaphy has fallen out of favor owing to concern for massive chondrolysis, a devastating consequence that is believed to outweigh any potential benefit.^17^^,^^34^^,^^48^^,^^50^^,^^63^

### Open anterior-inferior capsular shift

**Figure 5:**
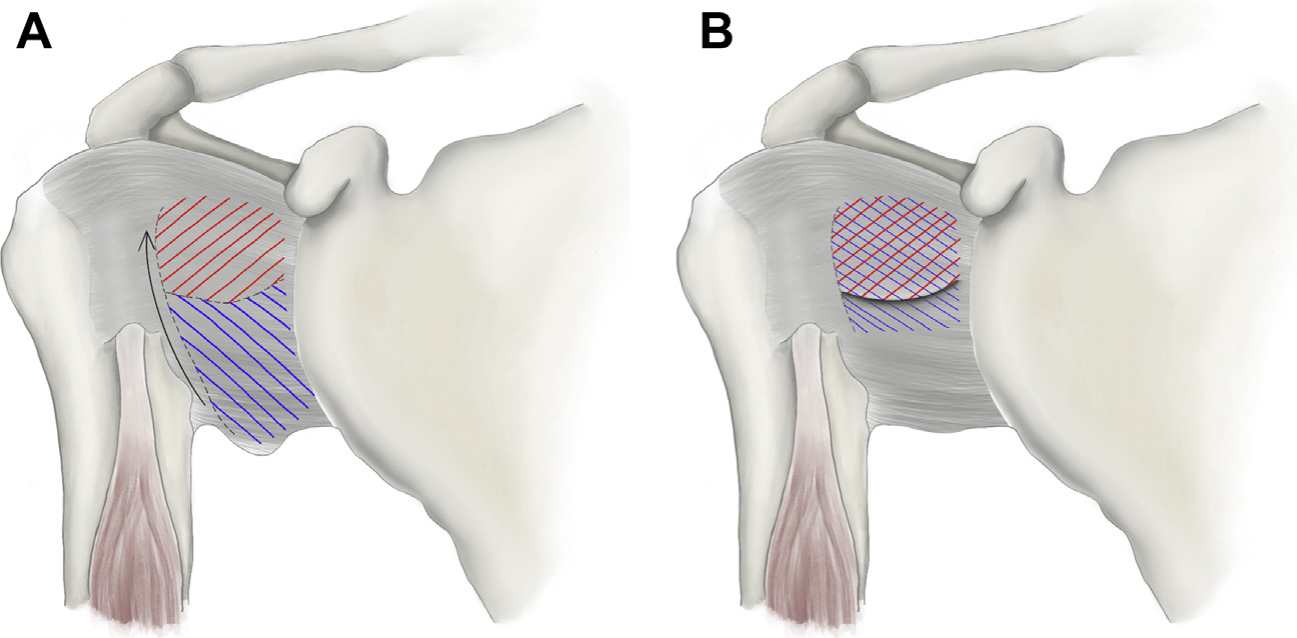
Illustration of Neer open capsular shift demonstrating the creation of two flaps using a T-incision (**A**) and tightening of the capsule via overlap of the flaps to provide reinforcement (**B**).

Initially described by Neer and Foster^57^ in 1980 for involuntary inferior and multidirectional shoulder instability, the open inferior capsular shift has represented the gold standard in the treatment of patients with MDI. The authors recognized that most approaches in these patients not only inadequately treat the redundant inferior capsule but also can cause a fixed subluxation or dislocation opposite to the direction of the repair. Thus, this procedure detaches the capsule from the humeral neck in two flaps, followed by proximally shifting the flaps, creating an overlap that reinforces the anterior capsular laxity and reduces the volume of the redundant inferior capsular pouch (Fig. 5). In their initial article, Neer and Foster^57^ noted only 1 unsatisfactory result of 40 shoulders with MDI, including 17 shoulders with greater than two years of follow-up. Pollack et al^64^ used this same technique in a larger cohort of 49 patients with MDI with an average of five years of follow-up. Forty-seven (96%) shoulders were stable at the time of the final follow-up, and 46 shoulders had either good or excellent results.

Although these articles report impressive outcomes in shoulders with MDI that were refractory to conservative measures, there was no specific mention of EDS, making it difficult to extrapolate these results to a population with underlying connective tissue disease. In patients with EDS, the inferior and anterior capsule has very little structural integrity, making capsular shifts potentially more difficult or less effective. A 2016 study investigated outcomes of the Neer open inferior capsular shift technique in 15 adolescent patients with generalized ligamentous hyperlaxity.^85^ All patients had a Beighton score of >6, with a positive genetic test for EDS in five participants, although it should be noted that there is no genetic marker for EDS subtypes including hEDS. Thirteen of the 15 patients experienced improvement in their pain, and an identical percentage reported an improvement in stability.

### Open capsular reconstruction

Reconstruction of the anterior capsule with a soft-tissue allograft or autograft has been described for treatment of instability in patients with collagen disorders or as a salvage procedure after multiple failed operations.^2^^,^^20^^,^^37^^,^^41^^,^^75^^,^^87^ The technique involves fixation of allograft or autograft tissue to the anterior glenoid rim to reconstruct the anterior labrum, with free ends of the graft used to reconstruct the middle and inferior glenohumeral ligaments.^10^ For patients with EDS, allografts should routinely be used, given the compromised soft-tissue integrity of autograft tendons or fascia.

The added anterior and inferior stability would theoretically confer a treatment benefit for the patient with EDS with anterior and anterior-inferior instability. However, outcomes in this population have been variable. In a retrospective series that included 10 shoulders in five patients with hEDS, anterior capsular reconstruction with tibialis anterior tendon allograft resulted in recurrence of shoulder instability in 60% of cases.^20^ A more recent report on five shoulders in four female patients with hEDS investigated outcomes for open capsular shift combined with anterior capsular reconstruction using Achilles tendon allograft.^75^ In four of five cases, the procedure resulted in lasting improvement in shoulder pain and stability. Chaudhury et al^15^ presented a more extensive allograft augmentation of both the anterior and posterior capsule using Achilles allograft in a patient with hEDS, producing lasting stability in both shoulders. Use of acellular dermal allograft in posterior capsular reconstruction has also been described in a patient with hEDS after prior failed capsulorrhaphy, though results have not been reported.^41^

### Latarjet or bone block procedures

Bony augmentation procedures have been suggested as a way to expand the glenohumeral articular interface and decrease reliance on the abnormal native soft tissue seen in patients with hEDS. The coracoid graft in the Latarjet procedure extends the articulating glenoid arc; it also provides additional dynamic stability in abduction and external rotation via the conjoint tendon compressing the inferior subscapularis. However, success of the Latarjet procedure in MDI and generalized hypermobility is limited to those with predominantly anterior instability.^1^^,^^42^ In a patient with true MDI and equal anterior and posterior instability symptoms, the Latarjet theoretically could correct the anterior instability, while inducing more pronounced posterior instability.^21^

Glenoid augmentation with iliac crest autograft has been shown to be effective in patients with traumatic recurrent instability and glenoid deficiency.^88^ Although traditionally performed via an open technique, Armstrong et al^4^ described an arthroscopic technique combining capsular plication with iliac crest autograft in a patient with EDS and MDI. This approach is minimally invasive and preserves the coracoid-conjoint complex, but harvest site morbidity may be a concern for patients with EDS who are prone to wound complications. This procedure is also technically difficult, given that the conjoint tendon remains intact, often blocking the normal angle for screw placement in this procedure.

Distal tibial allograft provides additional articular surface area with similar outcomes to the Latarjet procedure.^27^ Distal tibial allograft provides an option for those who have failed the Latarjet procedure and avoids graft site morbidity,^62^ though outcomes in patients with hEDS have yet to be described in the literature.

### Glenoid osteotomy

For patients with hEDS with a dysplastic or retroverted glenoid, open wedge glenoid osteotomy may be considered. While there are no published cases of glenoid osteotomy performed in a patient with hEDS, correction of glenoid version has produced reliable results in healthy patients with posterior shoulder instability and a retroverted glenoid.^7^^,^^46^^,^^65^ Hypermobile patients with EDS with dysplastic glenoids may therefore benefit from osteotomy in conjunction with a procedure that reduces the size and reinforces the integrity of the glenohumeral capsule.

### Salvage procedures

Unfortunately, the challenges in treatment of hEDS can result in years of pain and functional debilitation, despite months of physical therapy and multiple attempts at surgery. For those who continue to experience frequent dislocations or have evidence of severe joint deterioration, shoulder arthrodesis has been used as a salvage procedure.^47^ However, in the senior author’s opinion, this should not be performed until every other option has been used, as weakened scapulothoracic musculature can cause marked pain and discomfort, transferring it from the glenohumeral joint to the scapulothoracic articulations. Reverse total shoulder arthroplasty with proper tensioning may be a more reasonable salvage option in older patients with EDS. The literature on shoulder arthroplasty in the setting of hEDS is limited to a single case report with an unsatisfactory outcome owing to development of complex scapular winging.^78^ When considering reverse total shoulder arthroplasty in this population, it carries a higher risk owing to potential for dislocation and therefore should be approached with caution.

### Author’s preferred technique

**Figure 6:**
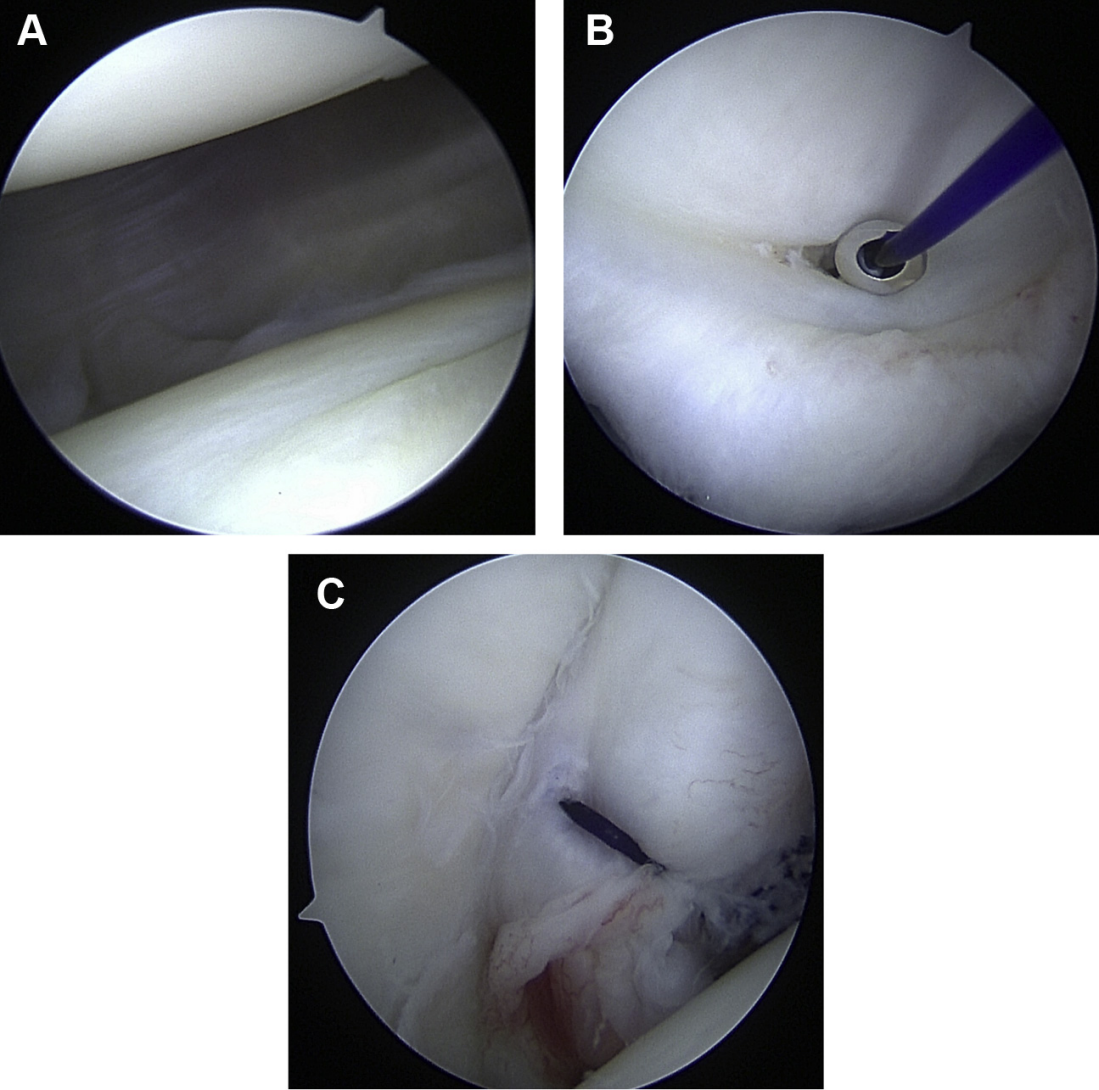
Arthroscopic capsulorrhaphy using PDS suture to reduce capsular laxity.

The senior author’s preferred treatment in patients with hEDS and associated hypermobility involves a 270º capsulorrhaphy, involving the anterior, inferior, and posterior capsule. In this technique, the capsulorrhaphy is performed using absorbable PDS sutures without anchors (Fig. 6). This has the advantage of not only tightening the capsule but as the sutures resorb, they potentially create an inflammatory reaction that can beneficially stiffen the capsule through scarring.^76^ It also avoids the need for permanent anchors to be placed in young patients with otherwise healthy glenoid articular surfaces. The patient is positioned in either the beach-chair or lateral position. After creating the standard posterior viewing portal in line with the joint line, two anterior portals are created via the percutaneous cannula kits – one immediately above the subscapularis tendon in line with the joint and a second just anterior to the supraspinatus high in the rotator interval. After cannulating both portals, these are used as the primary working portals for the anterior and inferior capsulorrhaphy. However, the senior author’s preference is to start posteriorly to avoid closing down the joint if starting anteriorly, so the viewing portal is switched to the anterosuperior portal. Multiple figure of eight and simple sutures are placed using curved and angled suture passing device in the posterior capsule from 10 o’clock to 6 o’clock, using the posterior portal to pass and tie the sutures, while the anteroinferior portal as the accessory working portal. The sutures are placed and tied from inferior to superior. Next, using a switching stick, the camera is changed back to the posterior portal. Through the inferoanterior portal, multiple figure of eight and simple sutures are then placed using the curved and angled suture passer. The sutures are placed and tied inferiorly to superiorly, from the 6 o’clock to the 2 o’clock positions. Depending on the residual laxity, we often place two rotator interval stiches between the subscapularis and the supraspinatus. The patient is then immobilized for a minimum of 6 weeks, starting active range of motion exercises once there is no residual sulcus sign on physical examination in the clinic. Stretching is not permitted until 12 weeks postoperatively. Strengthening is begun between 12 and 16 weeks, and return to most sports will start after 16 weeks.

## Overview

Shoulder instability is common in hEDS and can result in lifelong pain and functional impairment. Treatment in this population is complicated by the severe degree of instability as well as the underlying abnormalities of the joint connective tissue. Physical therapy is the mainstay of treatment and should focus on dynamic kinetics, resting rotator cuff tone, and scapulothoracic mechanics. Patients who fail conservative management may require attempts at surgical stabilization with the understanding that outcomes in this population tend to be worse than healthy patients with MDI. Operative treatment should focus on addressing capsular redundancy and reinforcement with allograft tissue. Bone block procedures may be reserved for those who have recurrent stability after initial stabilization attempts. Salvage options such as arthroplasty and arthrodesis are reserved for those who fail multiple surgical treatments. These procedures should be performed by a surgeon who is familiar with hypermobile patients owing to the likelihood and nature of ensuing complications. Given the relative rarity of hEDS, there is a scarcity of reported outcomes for approaches to both operative and nonoperative management. The challenging management of these patients should therefore be individualized with careful consideration of the available literature.

## Disclaimers

Michael Gottschalk reports being a PI for Stryker, Arthrex, Acumed, Konica Minolta and a consultant for Stryker and Wright.

Eric Wagner reports being a consultant for Stryker and recieved grant from Arthrex and DJO.

The other authors, their immediate family, and any research foundation with which they are affiliated did not receive any financial payments or other benefits from any commercial entity related to the subject of this article.

Funding: No funding was disclosed by the author(s).
